# 2,2-Diethyl 3,4-dimethyl 5-(4-cyano­phen­yl)pyrrolidine-2,2,3,4-tetra­carboxyl­ate

**DOI:** 10.1107/S1600536812029625

**Published:** 2012-07-07

**Authors:** Long He

**Affiliations:** a College of Chemistry and Chemical Engineering, China West Normal University, Nanchong 637002, People’s Republic of China

## Abstract

The title compound, C_21_H_24_N_2_O_8_, was synthesized by a 1,3-dipolar cyclo­addition reaction of dimethyl fumarate, diethyl 2-amino­malonate and 4-cyano­benzaldehyde. Both methyl ester groups display a *trans* configuration and the pyrrolidine ring possesses an envelope conformation, with the C atom in the 3-position as the flap. In the crystal, N—H⋯N hydrogen bonds and weak C—H⋯O inter­actions occur.

## Related literature

For the biological activity of pyrrolidine derivatives, see: Coldham & Hufton (2005 ▶); Pandey *et al.* (2006 ▶); Schreiber *et al.* (2000 ▶). For a related structure, see: He *et al.* (2010 ▶).
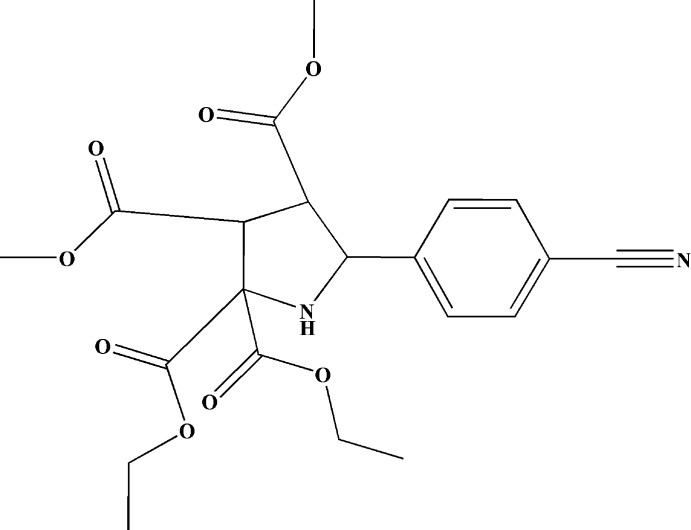


## Experimental

### 

#### Crystal data

C_21_H_24_N_2_O_8_
*M* *_r_* = 432.42
Orthorhombic, 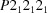

*a* = 8.4720 (2) Å
*b* = 10.3043 (2) Å
*c* = 25.8774 (5) Å
*V* = 2259.05 (8) Å^3^
*Z* = 4
Cu *K*α radiation
μ = 0.83 mm^−1^
*T* = 291 K
0.38 × 0.30 × 0.30 mm

#### Data collection

Oxford Diffraction Gemini S Ultra diffractometer
Absorption correction: multi-scan (*CrysAlis PRO*; Oxford Diffraction, 2007 ▶) *T*_min_ = 0.744, *T*_max_ = 0.789
26556 measured reflections
2565 independent reflections
2050 reflections with *I* > 2σ(*I*)
*R*_int_ = 0.027

#### Refinement

*R*[*F* ^2^ > 2σ(*F* ^2^)] = 0.050
*wR*(*F* ^2^) = 0.141
*S* = 1.03
2565 reflections
288 parameters
2 restraints
H atoms treated by a mixture of independent and constrained refinement
Δρ_max_ = 0.40 e Å^−3^
Δρ_min_ = −0.22 e Å^−3^

### 

Data collection: *CrysAlis PRO* (Oxford Diffraction, 2007 ▶); cell refinement: *CrysAlis PRO*; data reduction: *CrysAlis PRO*; program(s) used to solve structure: *SHELXS97* (Sheldrick, 2008 ▶); program(s) used to refine structure: *SHELXL97* (Sheldrick, 2008 ▶); molecular graphics: *ORTEP-3* (Farrugia, 1997 ▶); software used to prepare material for publication: *SHELXL97*.

## Supplementary Material

Crystal structure: contains datablock(s) global, I. DOI: 10.1107/S1600536812029625/xu5557sup1.cif

Structure factors: contains datablock(s) I. DOI: 10.1107/S1600536812029625/xu5557Isup2.hkl

Supplementary material file. DOI: 10.1107/S1600536812029625/xu5557Isup3.cml

Additional supplementary materials:  crystallographic information; 3D view; checkCIF report

## Figures and Tables

**Table 1:** Hydrogen-bond geometry (Å, °)

*D*—H⋯*A*	*D*—H	H⋯*A*	*D*⋯*A*	*D*—H⋯*A*
N2—H1⋯N1^i^	0.86 (4)	2.47 (4)	3.240 (5)	148 (4)
C8—H8⋯O1^ii^	0.98	2.39	3.333 (4)	161 (1)
C13—H13*C*⋯O7^ii^	0.96	2.46	3.388 (6)	163
C18—H18*C*⋯O5^iii^	0.96	2.55	3.453 (9)	157

Symmetry codes: (i) 
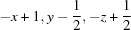
; (ii) 
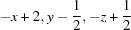
; (iii) 
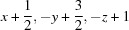
.

**Fig. 1.**
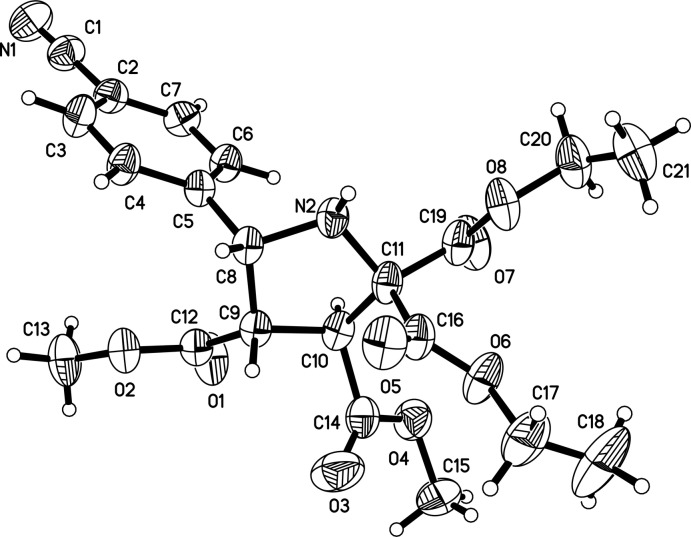
The molecular structure of (I) with 30% probability displacement ellipsoids (arbitrary spheres for H atoms).

## supplementary crystallographic information

### Comment

Facile synthesis of structurally diverse heterocycles is important in chemical biology. Five-membered pyrrolidines compounds is an important class of heterocyclic compounds with wide spread applications to the synthesis of biologically active compounds and natural alkaloids (Coldham & Hufton, 2005; Pandey *et al.*, 2006; Schreiber *et al.*, 2000). Its crystal structure is reported here.

The molecular structure of (I) is shown in Fig. 1. Bond lengths and angles in (I) are normal. The pyrrolidine ring possesses an envelope conformation. The crystal packing is stabilized by C—H···O and N—H···N hydrogen bonds (Table 1).

### Experimental

To a solution of 4-cyanobenzaldehyde (0.065 g, 0.5 mmol), sodium sulflate (0.1 g) and diethyl 2-aminomalonate (0.070 g, 0.4 mmol) in chloroform (4 ml) was added dimethyl fumarate (0.145 g, 1 mmol). The mixture was stirred at 323 K for 2 d and then cooled to room temperature, the solvent was evaporated under reduced pressure and the residues was purified by flash chromatograghy on silica gel. The colourless powder was obtained. Single crystals suitable for X-ray diffraction were obtained by slow evaporation of an ethyl acetate solution.

### Refinement

H atoms on N atoms were located in a difference Fourier map and refined isotropically. The carbon-bound hydrogen atoms were placed in calculated positions, with C—H = 0.93–0.98 Å, and refined using a riding model, with *U*_iso_(H) =1.5*U*_eq_(C) for methyl H atoms and *U*_iso_(H) =1.2*U*_eq_(C) for the others. As no significant anomalous scatterings Friedel pairs were merged.

### Crystal data

**Table:**

C_21_H_24_N_2_O_8_	*D*_x_ = 1.271 Mg m^−^^3^
*M**_r_* = 432.42	Cu *K*α radiation, λ = 1.54184 Å
Orthorhombic, *P*2_1_2_1_2_1_	Cell parameters from 10771 reflections
*a* = 8.4720 (2) Å	θ = 1.7–72.3°
*b* = 10.3043 (2) Å	µ = 0.83 mm^−^^1^
*c* = 25.8774 (5) Å	*T* = 291 K
*V* = 2259.05 (8) Å^3^	Block, colorless
*Z* = 4	0.38 × 0.30 × 0.30 mm
*F*(000) = 912	

### Data collection

**Table:**

Oxford Diffraction Gemini S Ultra diffractometer	2565 independent reflections
Radiation source: Enhance Ultra (Cu) X-ray Source	2050 reflections with *I* > 2σ(*I*)
Mirror monochromator	*R*_int_ = 0.027
Detector resolution: 15.9149 pixels mm^-1^	θ_max_ = 72.6°, θ_min_ = 3.4°
ω scans	*h* = −10→10
Absorption correction: multi-scan (*CrysAlis PRO*; Oxford Diffraction, 2007)	*k* = −12→10
*T*_min_ = 0.744, *T*_max_ = 0.789	*l* = −31→31
26556 measured reflections	

### Refinement

**Table:**

Refinement on *F*^2^	Secondary atom site location: difference Fourier map
Least-squares matrix: full	Hydrogen site location: inferred from neighbouring sites
*R*[*F*^2^ > 2σ(*F*^2^)] = 0.050	H atoms treated by a mixture of independent and constrained refinement
*wR*(*F*^2^) = 0.141	*w* = 1/[σ^2^(*F*_o_^2^) + (0.0724*P*)^2^ + 0.5988*P*] where *P* = (*F*_o_^2^ + 2*F*_c_^2^)/3
*S* = 1.03	(Δ/σ)_max_ < 0.001
2565 reflections	Δρ_max_ = 0.40 e Å^−^^3^
288 parameters	Δρ_min_ = −0.22 e Å^−^^3^
2 restraints	Extinction correction: *SHELXL97* (Sheldrick, 2008), Fc^*^=kFc[1+0.001xFc^2^λ^3^/sin(2θ)]^-1/4^
Primary atom site location: structure-invariant direct methods	Extinction coefficient: 0.0015 (4)

### Special details

**Table:**

Geometry. All e.s.d.'s (except the e.s.d. in the dihedral angle between two l.s. planes) are estimated using the full covariance matrix. The cell e.s.d.'s are taken into account individually in the estimation of e.s.d.'s in distances, angles and torsion angles; correlations between e.s.d.'s in cell parameters are only used when they are defined by crystal symmetry. An approximate (isotropic) treatment of cell e.s.d.'s is used for estimating e.s.d.'s involving l.s. planes.
Refinement. Refinement of *F*^2^ against ALL reflections. The weighted *R*-factor *wR* and goodness of fit *S* are based on *F*^2^, conventional *R*-factors *R* are based on *F*, with *F* set to zero for negative *F*^2^. The threshold expression of *F*^2^ > σ(*F*^2^) is used only for calculating *R*-factors(gt) *etc*. and is not relevant to the choice of reflections for refinement. *R*-factors based on *F*^2^ are statistically about twice as large as those based on *F*, and *R*- factors based on ALL data will be even larger.

### Fractional atomic coordinates and isotropic or equivalent isotropic displacement parameters (Å^2^)

**Table:**

	*x*	*y*	*z*	*U*_iso_*/*U*_eq_	
O2	1.0668 (3)	0.9425 (2)	0.20700 (8)	0.0707 (7)	
O5	1.0369 (4)	0.7873 (2)	0.40309 (11)	0.0880 (9)	
O8	0.8498 (4)	1.0544 (3)	0.46182 (9)	0.0865 (9)	
N2	0.8356 (4)	0.9769 (3)	0.35733 (10)	0.0622 (7)	
O1	1.1491 (5)	1.1320 (3)	0.23785 (10)	0.0928 (10)	
C9	1.0464 (4)	0.9727 (3)	0.29624 (11)	0.0548 (7)	
H9	1.1073	0.8930	0.3016	0.066*	
O6	1.1628 (4)	0.9441 (3)	0.44645 (10)	0.0898 (9)	
O4	1.3158 (4)	1.1691 (3)	0.37339 (11)	0.0874 (8)	
O7	0.9598 (5)	1.2258 (3)	0.42249 (12)	0.1046 (12)	
C5	0.7612 (4)	1.0072 (3)	0.26561 (11)	0.0542 (7)	
C2	0.5727 (4)	1.1296 (3)	0.19214 (13)	0.0632 (9)	
C8	0.8696 (4)	0.9382 (3)	0.30325 (11)	0.0530 (7)	
H8	0.8559	0.8442	0.2997	0.064*	
C12	1.0926 (4)	1.0266 (3)	0.24488 (12)	0.0597 (8)	
C6	0.7360 (4)	1.1396 (3)	0.26702 (13)	0.0620 (8)	
H6	0.7825	1.1885	0.2931	0.074*	
C10	1.0857 (4)	1.0649 (3)	0.34090 (12)	0.0592 (8)	
H10	1.0539	1.1535	0.3320	0.071*	
C4	0.6885 (5)	0.9354 (4)	0.22681 (13)	0.0668 (9)	
H4	0.7047	0.8462	0.2251	0.080*	
C11	0.9802 (4)	1.0124 (3)	0.38427 (12)	0.0602 (8)	
C7	0.6433 (4)	1.2009 (3)	0.23057 (14)	0.0658 (9)	
H7	0.6287	1.2903	0.2320	0.079*	
C19	0.9328 (5)	1.1127 (3)	0.42459 (13)	0.0716 (10)	
C16	1.0612 (5)	0.8987 (3)	0.41186 (13)	0.0683 (10)	
C3	0.5921 (5)	0.9951 (4)	0.19062 (15)	0.0754 (11)	
H3	0.5409	0.9460	0.1656	0.091*	
C14	1.2688 (5)	1.0600 (4)	0.35092 (14)	0.0719 (10)	
C13	1.0929 (7)	0.9876 (5)	0.15484 (13)	0.0955 (15)	
H13A	1.2035	1.0033	0.1497	0.115*	
H13B	1.0352	1.0665	0.1493	0.115*	
H13C	1.0575	0.9228	0.1308	0.115*	
C20	0.7915 (8)	1.1323 (5)	0.50465 (15)	0.1021 (16)	
H20A	0.8668	1.2001	0.5128	0.122*	
H20B	0.6923	1.1729	0.4952	0.122*	
C17	1.2540 (8)	0.8528 (5)	0.47718 (19)	0.1127 (19)	
H17A	1.1848	0.7871	0.4916	0.135*	
H17B	1.3318	0.8100	0.4556	0.135*	
C15	1.4850 (5)	1.1640 (7)	0.3860 (2)	0.1114 (17)	
H15A	1.5435	1.1382	0.3559	0.167*	
H15B	1.5020	1.1022	0.4132	0.167*	
H15C	1.5198	1.2481	0.3971	0.167*	
C21	0.7690 (9)	1.0492 (6)	0.54894 (18)	0.121 (2)	
H21A	0.8670	1.0078	0.5575	0.145*	
H21B	0.6912	0.9845	0.5410	0.145*	
H21C	0.7337	1.1003	0.5778	0.145*	
C18	1.3299 (11)	0.9207 (8)	0.5172 (3)	0.189 (4)	
H18A	1.2602	0.9860	0.5306	0.227*	
H18B	1.4238	0.9610	0.5040	0.227*	
H18C	1.3575	0.8613	0.5443	0.227*	
O3	1.3504 (4)	0.9759 (4)	0.33931 (18)	0.1249 (14)	
N1	0.4052 (5)	1.2386 (4)	0.12122 (16)	0.0982 (12)	
C1	0.4786 (5)	1.1909 (4)	0.15304 (15)	0.0752 (10)	
H1	0.780 (5)	0.924 (4)	0.3757 (16)	0.113*	

### Atomic displacement parameters (Å^2^)

**Table:**

	*U*^11^	*U*^22^	*U*^33^	*U*^12^	*U*^13^	*U*^23^
O2	0.1013 (19)	0.0581 (13)	0.0527 (11)	−0.0096 (14)	0.0014 (12)	−0.0020 (11)
O5	0.126 (2)	0.0517 (14)	0.0863 (18)	0.0007 (16)	−0.0135 (18)	0.0065 (12)
O8	0.134 (3)	0.0700 (15)	0.0558 (12)	−0.0049 (18)	0.0108 (16)	−0.0103 (12)
N2	0.0743 (17)	0.0655 (17)	0.0469 (13)	−0.0087 (16)	−0.0029 (13)	0.0032 (12)
O1	0.148 (3)	0.0629 (16)	0.0677 (15)	−0.0344 (19)	0.0105 (18)	0.0011 (13)
C9	0.0710 (19)	0.0417 (15)	0.0516 (15)	0.0009 (15)	−0.0052 (15)	0.0015 (13)
O6	0.121 (2)	0.0700 (15)	0.0782 (16)	−0.0008 (18)	−0.0441 (17)	0.0122 (13)
O4	0.0833 (17)	0.094 (2)	0.0853 (17)	−0.0168 (17)	−0.0061 (16)	−0.0093 (16)
O7	0.170 (4)	0.0548 (16)	0.089 (2)	−0.0019 (19)	0.017 (2)	−0.0071 (13)
C5	0.0664 (18)	0.0482 (16)	0.0479 (15)	−0.0017 (15)	−0.0027 (14)	−0.0003 (13)
C2	0.0652 (19)	0.064 (2)	0.0606 (18)	0.0040 (18)	−0.0029 (16)	0.0074 (16)
C8	0.0706 (19)	0.0414 (14)	0.0472 (14)	−0.0014 (15)	−0.0051 (13)	0.0015 (13)
C12	0.074 (2)	0.0487 (16)	0.0569 (17)	0.0013 (16)	−0.0020 (16)	0.0020 (14)
C6	0.077 (2)	0.0460 (17)	0.0625 (18)	−0.0003 (17)	−0.0093 (17)	−0.0035 (15)
C10	0.078 (2)	0.0439 (15)	0.0556 (16)	−0.0060 (16)	−0.0124 (15)	0.0046 (14)
C4	0.085 (2)	0.0502 (17)	0.0648 (18)	0.0030 (18)	−0.0177 (19)	−0.0071 (16)
C11	0.088 (2)	0.0463 (16)	0.0465 (14)	−0.0032 (17)	−0.0107 (16)	0.0025 (13)
C7	0.069 (2)	0.0536 (18)	0.075 (2)	0.0036 (17)	−0.0033 (19)	0.0058 (17)
C19	0.106 (3)	0.0555 (19)	0.0537 (18)	0.001 (2)	−0.013 (2)	0.0007 (15)
C16	0.096 (3)	0.0555 (19)	0.0534 (17)	−0.0025 (19)	−0.0080 (19)	0.0078 (15)
C3	0.090 (3)	0.069 (2)	0.068 (2)	0.001 (2)	−0.024 (2)	−0.0083 (18)
C14	0.087 (3)	0.065 (2)	0.065 (2)	−0.011 (2)	−0.0043 (19)	−0.0079 (19)
C13	0.143 (4)	0.092 (3)	0.0511 (18)	−0.016 (3)	0.009 (2)	−0.0015 (19)
C20	0.147 (5)	0.094 (3)	0.064 (2)	0.014 (3)	0.006 (3)	−0.020 (2)
C17	0.140 (5)	0.094 (3)	0.104 (3)	0.014 (4)	−0.046 (4)	0.027 (3)
C15	0.064 (2)	0.148 (5)	0.122 (4)	−0.008 (3)	−0.014 (3)	−0.017 (4)
C21	0.158 (5)	0.123 (4)	0.081 (3)	−0.002 (4)	0.032 (3)	−0.016 (3)
C18	0.220 (9)	0.184 (7)	0.164 (6)	0.007 (7)	−0.125 (7)	0.040 (6)
O3	0.095 (2)	0.101 (2)	0.179 (4)	0.024 (2)	−0.037 (3)	−0.040 (3)
N1	0.092 (2)	0.103 (3)	0.099 (3)	0.012 (2)	−0.028 (2)	0.020 (2)
C1	0.070 (2)	0.080 (2)	0.075 (2)	0.004 (2)	−0.008 (2)	0.008 (2)

### Geometric parameters (Å, º)

**Table:**

O2—C12	1.327 (4)	C10—C14	1.573 (6)
O2—C13	1.445 (4)	C10—H10	0.9800
O5—C16	1.188 (4)	C4—C3	1.386 (5)
O8—C19	1.335 (5)	C4—H4	0.9300
O8—C20	1.455 (5)	C11—C19	1.523 (5)
N2—C11	1.456 (5)	C11—C16	1.534 (5)
N2—C8	1.484 (4)	C7—H7	0.9300
N2—H1	0.86 (2)	C3—H3	0.9300
O1—C12	1.201 (4)	C14—O3	1.149 (5)
C9—C12	1.493 (4)	C13—H13A	0.9600
C9—C10	1.532 (4)	C13—H13B	0.9600
C9—C8	1.550 (5)	C13—H13C	0.9600
C9—H9	0.9800	C20—C21	1.443 (7)
O6—C16	1.327 (5)	C20—H20A	0.9700
O6—C17	1.454 (5)	C20—H20B	0.9700
O4—C14	1.327 (5)	C17—C18	1.405 (8)
O4—C15	1.471 (5)	C17—H17A	0.9700
O7—C19	1.189 (5)	C17—H17B	0.9700
C5—C6	1.381 (4)	C15—H15A	0.9600
C5—C4	1.391 (5)	C15—H15B	0.9600
C5—C8	1.516 (4)	C15—H15C	0.9600
C2—C7	1.374 (5)	C21—H21A	0.9600
C2—C3	1.396 (5)	C21—H21B	0.9600
C2—C1	1.435 (5)	C21—H21C	0.9600
C8—H8	0.9800	C18—H18A	0.9600
C6—C7	1.380 (5)	C18—H18B	0.9600
C6—H6	0.9300	C18—H18C	0.9600
C10—C11	1.534 (5)	N1—C1	1.143 (5)
			
C12—O2—C13	117.1 (3)	O7—C19—O8	125.1 (4)
C19—O8—C20	118.7 (3)	O7—C19—C11	125.7 (4)
C11—N2—C8	110.8 (3)	O8—C19—C11	109.1 (3)
C11—N2—H1	111 (3)	O5—C16—O6	125.6 (4)
C8—N2—H1	117 (3)	O5—C16—C11	124.8 (4)
C12—C9—C10	112.6 (3)	O6—C16—C11	109.6 (3)
C12—C9—C8	116.3 (3)	C4—C3—C2	119.4 (4)
C10—C9—C8	105.3 (3)	C4—C3—H3	120.3
C12—C9—H9	107.4	C2—C3—H3	120.3
C10—C9—H9	107.4	O3—C14—O4	124.9 (4)
C8—C9—H9	107.4	O3—C14—C10	125.1 (4)
C16—O6—C17	119.1 (3)	O4—C14—C10	110.0 (4)
C14—O4—C15	111.1 (4)	O2—C13—H13A	109.5
C6—C5—C4	118.4 (3)	O2—C13—H13B	109.5
C6—C5—C8	122.7 (3)	H13A—C13—H13B	109.5
C4—C5—C8	118.8 (3)	O2—C13—H13C	109.5
C7—C2—C3	120.0 (3)	H13A—C13—H13C	109.5
C7—C2—C1	121.1 (3)	H13B—C13—H13C	109.5
C3—C2—C1	118.9 (4)	C21—C20—O8	108.8 (4)
N2—C8—C5	111.2 (3)	C21—C20—H20A	109.9
N2—C8—C9	103.7 (3)	O8—C20—H20A	109.9
C5—C8—C9	113.7 (3)	C21—C20—H20B	109.9
N2—C8—H8	109.4	O8—C20—H20B	109.9
C5—C8—H8	109.4	H20A—C20—H20B	108.3
C9—C8—H8	109.4	C18—C17—O6	108.9 (5)
O1—C12—O2	123.0 (3)	C18—C17—H17A	109.9
O1—C12—C9	125.2 (3)	O6—C17—H17A	109.9
O2—C12—C9	111.8 (3)	C18—C17—H17B	109.9
C7—C6—C5	121.5 (3)	O6—C17—H17B	109.9
C7—C6—H6	119.3	H17A—C17—H17B	108.3
C5—C6—H6	119.3	O4—C15—H15A	109.5
C9—C10—C11	101.9 (3)	O4—C15—H15B	109.5
C9—C10—C14	108.6 (3)	H15A—C15—H15B	109.5
C11—C10—C14	116.3 (3)	O4—C15—H15C	109.5
C9—C10—H10	109.9	H15A—C15—H15C	109.5
C11—C10—H10	109.9	H15B—C15—H15C	109.5
C14—C10—H10	109.9	C20—C21—H21A	109.5
C3—C4—C5	120.8 (3)	C20—C21—H21B	109.5
C3—C4—H4	119.6	H21A—C21—H21B	109.5
C5—C4—H4	119.6	C20—C21—H21C	109.5
N2—C11—C19	106.1 (3)	H21A—C21—H21C	109.5
N2—C11—C10	103.2 (2)	H21B—C21—H21C	109.5
C19—C11—C10	114.6 (3)	C17—C18—H18A	109.5
N2—C11—C16	114.0 (3)	C17—C18—H18B	109.5
C19—C11—C16	108.5 (3)	H18A—C18—H18B	109.5
C10—C11—C16	110.4 (3)	C17—C18—H18C	109.5
C2—C7—C6	119.8 (3)	H18A—C18—H18C	109.5
C2—C7—H7	120.1	H18B—C18—H18C	109.5
C6—C7—H7	120.1	N1—C1—C2	178.8 (5)
			
C11—N2—C8—C5	−130.9 (3)	C3—C2—C7—C6	1.3 (6)
C11—N2—C8—C9	−8.3 (3)	C1—C2—C7—C6	−178.6 (4)
C6—C5—C8—N2	48.9 (4)	C5—C6—C7—C2	0.6 (6)
C4—C5—C8—N2	−133.6 (3)	C20—O8—C19—O7	−2.4 (7)
C6—C5—C8—C9	−67.7 (4)	C20—O8—C19—C11	−179.8 (4)
C4—C5—C8—C9	109.8 (4)	N2—C11—C19—O7	−106.8 (5)
C12—C9—C8—N2	−142.0 (3)	C10—C11—C19—O7	6.4 (6)
C10—C9—C8—N2	−16.5 (3)	C16—C11—C19—O7	130.3 (5)
C12—C9—C8—C5	−21.1 (4)	N2—C11—C19—O8	70.6 (4)
C10—C9—C8—C5	104.4 (3)	C10—C11—C19—O8	−176.2 (3)
C13—O2—C12—O1	−6.8 (6)	C16—C11—C19—O8	−52.3 (4)
C13—O2—C12—C9	174.4 (4)	C17—O6—C16—O5	−0.3 (7)
C10—C9—C12—O1	−2.6 (6)	C17—O6—C16—C11	−179.8 (4)
C8—C9—C12—O1	119.0 (4)	N2—C11—C16—O5	18.0 (6)
C10—C9—C12—O2	176.1 (3)	C19—C11—C16—O5	136.0 (4)
C8—C9—C12—O2	−62.2 (4)	C10—C11—C16—O5	−97.7 (5)
C4—C5—C6—C7	−1.1 (6)	N2—C11—C16—O6	−162.5 (3)
C8—C5—C6—C7	176.5 (3)	C19—C11—C16—O6	−44.5 (4)
C12—C9—C10—C11	161.4 (3)	C10—C11—C16—O6	81.8 (4)
C8—C9—C10—C11	33.7 (3)	C5—C4—C3—C2	2.3 (6)
C12—C9—C10—C14	−75.3 (4)	C7—C2—C3—C4	−2.7 (6)
C8—C9—C10—C14	157.0 (3)	C1—C2—C3—C4	177.1 (4)
C6—C5—C4—C3	−0.5 (6)	C15—O4—C14—O3	−5.2 (7)
C8—C5—C4—C3	−178.1 (4)	C15—O4—C14—C10	176.6 (4)
C8—N2—C11—C19	150.5 (3)	C9—C10—C14—O3	−22.5 (6)
C8—N2—C11—C10	29.7 (3)	C11—C10—C14—O3	91.7 (5)
C8—N2—C11—C16	−90.1 (3)	C9—C10—C14—O4	155.7 (3)
C9—C10—C11—N2	−38.3 (3)	C11—C10—C14—O4	−90.1 (4)
C14—C10—C11—N2	−156.2 (3)	C19—O8—C20—C21	−155.3 (5)
C9—C10—C11—C19	−153.2 (3)	C16—O6—C17—C18	−169.0 (6)
C14—C10—C11—C19	88.9 (4)	C7—C2—C1—N1	126 (23)
C9—C10—C11—C16	83.9 (3)	C3—C2—C1—N1	−54 (23)
C14—C10—C11—C16	−34.0 (4)		

### Hydrogen-bond geometry (Å, º)

**Table:**

*D*—H···*A*	*D*—H	H···*A*	*D*···*A*	*D*—H···*A*
N2—H1···N1^i^	0.86 (4)	2.47 (4)	3.240 (5)	148 (4)
C8—H8···O1^ii^	0.98	2.39	3.333 (4)	161 (1)
C13—H13*C*···O7^ii^	0.96	2.46	3.388 (6)	163
C18—H18*C*···O5^iii^	0.96	2.55	3.453 (9)	157

Symmetry codes: (i) −*x*+1, *y*−1/2, −*z*+1/2; (ii) −*x*+2, *y*−1/2, −*z*+1/2; (iii) *x*+1/2, −*y*+3/2, −*z*+1.

## References

[bb1] Coldham, I. & Hufton, R. (2005). *Chem. Rev.* **105**, 2765–2810. 10.1021/cr040004c 16011324

[bb2] Farrugia, L. J. (1997). *J. Appl. Cryst.* **30**, 565.

[bb3] He, L. (2010). *Acta Cryst.* E**66**, o3205.

[bb4] Oxford Diffraction (2007). *CrysAlis PRO* Oxford Diffraction Ltd, Abingdon, England.

[bb5] Pandey, G., Banerjee, P. & Gadre, S. R. (2006). *Chem. Rev.* **106**, 4484–4517. 10.1021/cr050011g 17091927

[bb6] Schreiber, S. L. (2000). *Science*, **287**, 1964–1969. 10.1126/science.287.5460.1964 10720315

[bb7] Sheldrick, G. M. (2008). *Acta Cryst.* A**64**, 112–122. 10.1107/S0108767307043930 18156677

